# Exploring person‐centred fundamental nursing care in hospital wards: A multi‐site ethnography

**DOI:** 10.1111/jocn.15024

**Published:** 2019-09-08

**Authors:** Elise van Belle, Jeltje Giesen, Tiffany Conroy, Marloes van Mierlo, Hester Vermeulen, Getty Huisman‐de Waal, Maud Heinen

**Affiliations:** ^1^ Radboud Institute for Health Sciences IQ Healthcare Radboud University Medical Center Nijmegen The Netherlands; ^2^ Department of Cardiology Radboud University Medical Center Nijmegen The Netherlands; ^3^ College of Nursing and Health Sciences Flinders University Bedford Park South Australia Australia; ^4^ Clinical Research Department Rijnstate Hospital Arnhem The Netherlands; ^5^ Faculty of Health and Social Studies HAN University of Applied Sciences Nijmegen The Netherlands

**Keywords:** acute care, clinical reasoning, ethnography, fundamentals of care, hospital, nursing, observation, patient participation, person‐centred care

## Abstract

**Objective:**

To explore how nurses in hospitals enact person‐centred fundamental care delivery.

**Background:**

Effective person‐centred care is at the heart of fundamental nursing care, but it is deemed to be challenging in acute health care as there is a strong biomedical focus and most nurses are not trained in person‐centred fundamental care delivery. We therefore need to know if and how nurses currently incorporate a person‐centred approach during fundamental care.

**Design:**

Focused ethnography approach.

**Methods:**

Observations of 30 nurses on three different wards in two Dutch hospitals during their morning shift. Data were collected through passive observations and analysed using framework analysis based on the fundamentals of care framework. The COREQ guideline was used for reporting.

**Results:**

Some nurses successfully integrate physical, psychosocial and relational elements of care in patient interactions. However, most nurses were observed to be mainly focused on physical care and did not take the time at their patients’ bedside to care for their psychosocial and relational needs. Many had a task‐focused way of working and communicating, seldom incorporating patients’ needs and experiences or discussing care planning, and often disturbing each other.

**Conclusions:**

This study demonstrates that although some nurses manage to do so, person‐centred fundamental care delivery remains a challenge in hospitals, as most nurses have a task‐focused approach and therefore do not manage to integrate the physical, relational and physical elements of care. For further improvement, attention needs to be paid to integrated fundamental care and clinical reasoning skills.

**Relevance to clinical practice:**

Although most nurses have a compassionate approach, this study shows that nurses do not incorporate psychosocial care or encourage patient participation when helping patients with their physical fundamental care needs, even though there seems to be sufficient opportunity for them to do so.

## 1 INTRODUCTION

Meeting the fundamental care needs of patients is essential for optimal safety, recovery and positive experiences within any healthcare setting (Kitson, Conroy, Wengstrom, Profetto‐McGrath, & Robertson‐Malt, 2010). While fundamental care is not a new concept, increasing attention is being placed on the ways in which it is delivered in practice (Richards, Hilli, Pentecost, Goodwin, & Frost, 2018; Zwakhalen et al., 2018). Fundamental nursing care is deeply entwined with person‐centred care, which has become the cornerstone of quality health care in many developed countries and is explicitly referenced in healthcare policies (Balik, Conway, Zipperer, & Watson, 2011; World Health Organization, 2000) and in Huber's holistic vision on health (Huber et al., 2011). Person‐centred care focuses on health care that involves patients through giving them greater influence in decision‐making and choice, and which is sensitive to the patients’ unique physical, psychosocial, cultural and emotional needs (Kitson, Marshall, Bassett, & Zeitz, 2013). The literature demonstrates that person‐centred care has the potential to reduce the length of hospital stays and to positively influence the maintenance of patients’ functional performance (Ekman et al., 2012). However, enacting person‐centred fundamental nursing care remains challenging.

## 2 BACKGROUND

Kitson et al. (2010) have defined the fundamentals of care (FoC) as the basic elements of nursing care. They encompass the physical, psychosocial and relational elements of care, and are required for every patient regardless of the patient's clinical condition or setting. The FoC Framework (FoCF) was developed to demonstrate how the FoC is related to the nurse–patient relationship and the care setting in which nursing care is to be delivered (Feo et al., 2018; Kitson, Conroy, Kaluski, Locock, & Lyons, 2013). At the core of the FoCF lies the nurse–patient relationship, which is essential for effective nursing care. The nurse–patient relationship is about approaching the patient in an individual way; it consists of developing trust with the patient, being able to focus on the patient, giving the patient undivided attention, anticipating the patient's needs and concerns, getting to know the patient and evaluating the quality of the relationship (Feo, Rasmussen, Wiechula, Conroy, & Kitson, 2017). Nurses who successfully use these relational elements of care can work effectively to meet the patient's fundamental needs (Feo & Kitson, 2016). There is, however, little evidence on how nurses actually integrate the patient's fundamental care needs (van Achterberg, 2014). Although Feo et al. recently published a guideline with recommendations for the nurse–patient relationship, these recommendations still need to be tested on its validity and alignment with the other FoCF dimensions (Feo, Conroy, et al., 2017).

We know that delivering effective person‐centred fundamental care is complex, requiring nurses to take into account their patients’ unique experiences, wishes and abilities which all have to be integrated into a personalised care plan (Kitson, 2018). We also know that many of today's nurses are not sufficiently trained in fundamental care delivery (MacMillan, 2016) or how to effectively involve patients in their own care (Huisman‐de Waal, Feo, Vermeulen, & Heinen, 2018). Although nurses stress the importance of person‐centred care where patients can participate (Bolster & Manias, 2010; Tobiano, Bucknall, Marshall, Guinane, & Chaboyer, 2015), difficulties are apparent from research demonstrating that nurses overestimate the patient‐centredness of their care, as compared to the patients’ experiences (Papastavrou et al., 2016; Suhonen et al., 2012). In a study by Jangland, Teodorsson, Molander, and Muntlin Athlin (2017) on the patients’ perspective of care delivery in surgical hospital wards, it was found that the high‐tempo culture leads to patients not receiving optimal physical or emotional support. The literature suggests that the current acute healthcare setting does not enable effective patient participation (Tobiano, Marshall, Bucknall, & Chaboyer, 2016), as it has a strong biomedical focus and places little priority on fundamental, person‐centred nursing care (Feo & Kitson, 2016), making patient participation hard to achieve (Hoglund, Winblad, Arnetz, & Arnetz, 2010; Ringdal, Chaboyer, Ulin, Bucknall, & Oxelmark, 2017).

With the current focus on person‐centred and holistic care, and a growing population of elderly patients with complex health conditions (Feo & Kitson, 2016), we need to generate insights into how to improve person‐centred fundamental care. Therefore, this study's objective was to gain insights into daily practice, by investigating person‐centredness and patient participation in fundamental care delivery by nurses in hospitals as a first step.

## 3 METHODS

### 3.1 Design

A focused ethnographic approach, using the direct observation of care, was used to gain insights into person‐centredness and patient participation in fundamental care delivery in the hospital setting. Observation is considered integral to a focused ethnography, because it provides the best opportunity to view participants’ behaviour in the context of the real world (Cruz & Higginbottom, 2013). The researchers acted as passive participants, observing nurses in their daily work. The COREQ guideline (Tong, Sainsbury, & Craig, 2007) was used for reporting (see Appendix S1).

### 3.2 Setting

To obtain a broad insight, the study was conducted on three nursing wards in two hospitals in the Netherlands: a cardiology and a geriatric ward in a regional hospital, and a surgical ward in a university hospital. In the cardiology ward, adult patients were admitted with acute cardiac problems such as myocardial infarction or arrhythmias. Patients with chronic heart failure were also admitted. In the geriatric ward, older patients with acute medical problems were treated. The surgical ward was a neurosurgical and plastic surgery ward where adult patients were treated with conditions relating to the brain or spine. The ward also admitted patients undergoing reconstructive surgery. In all three wards, patients were cared for by registered nurses with a vocational or bachelor degree. In all the wards, a nurse‐to‐patient ratio of 1:4 was common during the day, and the maximum ratio was 1:10 in the evenings. Each room consisted of one to four beds.

### 3.3 Participants

To be included in the study, the registered nurses were selected on the basis of age, gender, level of education and work experience, in order to ensure that there was maximum variation. They were selected with the help of their manager, who provided the nurses with information on the study and asked for their permission to be observed. All the patients older than 18 were eligible for inclusion. Patients were excluded if they did not consent to be observed or if the nurse deemed that the patient was not suitable for observation because of cognitive impairment or severe distress. The nurses and patients were informed that care in general would be observed, but were not informed about the specific aim of the study in order to avoid bias.

### 3.4 Data collection

Observations were conducted at the start of the nurses’ morning shift. The morning was chosen because it is traditionally a time with substantial patient interaction and fundamental nursing care delivery, such as helping patients with eating and drinking, handing out medication, washing and mobilising patients. The observations lasted for a minimum of 2.5 hr, starting at the morning shift handover and ending after those 2.5 hr at a natural moment of choice for the researcher (e.g., waiting for ward rounds or a patient interaction to end). Observations were conducted by three trained researchers, one for each of the three different wards, so as to minimise researcher bias. Each researcher performed the observations on one ward and thus became familiar with the patient care, the ward and the nursing team. All the researchers were registered nurses and had or were finalising their master's degree in Nursing. The first researcher (EvB) was trained in qualitative research, and she co‐observed and co‐transcribed the first observation period of the other researchers (JG, LC) to ensure consistency.

Before starting the observations, an observation guide was developed based on an earlier work by Conroy (2017). This guide provided prompts for information about the observed events, such as the location, date, time and specification of basic care needs, and was used during the observations for recording field notes. As a verbatim description of the whole event was not feasible, code words and abbreviations were developed. The field notes were transcribed directly after each observation to capture as much as possible of what was observed. The transcripts consist of rich data describing the setting, the nurse's behaviour, the nurse's actions, communication and notes on perceptions of the nurse's attitude, and any nonverbal communication such as body posture or eye contact. To avoid observer bias, the transcripts were written as neutrally and objectively as possible without making judgements. The observers were trained to be aware of their own experience as a nurse, and they reflected on any bias or personal feelings they might have through reflective notes after each observation period and through discussions with each other. As an additional member check, the transcripts were provided to the observed nurses, and they were asked if they agreed with the transcript or if they wanted to add to or rectify any of its passages.

During the observations, the researchers wore nurse's scrubs to conform with the attire of the observed nurses and indicate their own background as nurses. The nurses were informed that the observations were designed as nonparticipatory ones to observe the nurse's natural behaviour without intervening, but if patient safety was endangered or there were high levels of distress by nurse or patient, the researcher would be able to assist. To increase rapport and decrease the self‐consciousness of nurses being observed, the researcher had informal chats with the nurses before and during the observations, unless the nurses were in the presence of a patient or doing so would disturb the nursing process. Patients and nurses were told that all actions would be followed, but that both could indicate if the patient needed privacy on which the researchers would step outside the curtain or room. Data collection stopped after saturation was reached.

### 3.5 Data analysis

Analysis was conducted through framework analysis using the FoCF (Feo et al., 2018). In addition, all the data were screened for additional codes and themes, also known as thematic analysis. First, the researcher read the transcripts to establish an overview of the data. Second, the FoCF was used as the initial coding framework, and open coding was used for the text fragments that did not fit into the categories. Third, categories were revised and subcategories were developed. Fourth and finally, the researchers searched for patterns, associations, concepts and explanations in the data. All the transcripts were analysed using ATLAS.ti (ATLAS.ti Scientific Software Development, 2018). Thematic and open coding was conducted independently by three researchers (EvB, MH and JG), with two researchers coding the transcripts for each ward. In the cases of the cardiology and surgical ward, one of the coding researchers had also collected the data to incorporate the lived experience or elaborate on context. The other researcher provided a neutral view. The data collector of the geriatrics ward was contacted to provide background information on the transcript when necessary. Codes were compared and differences discussed until consensus was reached. Memos were written during the coding process to capture impressions and help the identification of themes and patterns during analysis.

### 3.6 Ethical considerations

According to the Dutch national legislation and as judged by the CMO Arnhem‐Nijmegen, who was the local Medical Ethics Committee, the study (file nr 2017‐3244) is noninvasive and does not fall under the scope of the Medical Research Involving Human Subjects Act (WMO; Ministry of Health, 2016). According to this law, as no identifying information from patients was gathered, written consent was not necessary. Participants were explicitly informed and both the researcher and attending nurse asked for their permission. They were also notified of their right to withdraw. If either the researcher or nurse doubted the patient's cognitive state, the patient's close relative was asked to provide written informed consent. All the nurses provided written consent and approved of the transcripts in writing. Data were analysed anonymously and stored separately from the nurses’ personal information.

## 4 RESULTS

A total of 30 observation periods were conducted, with 10 periods on each ward. The observations at the surgical ward were carried out from July–August 2017, while the observations at the geriatric and cardiology wards took place from February–April 2018. The observation periods lasted for a minimum of 2.5 hr and a maximum of 3.0 hr, and they all occurred between 7:30–11:00 a.m.

### 4.1 Nurses

All 30 nurses who were approached consented to be observed and checked the transcripts. No changes had to be made, as all the nurses approved the transcripts. According to the wards’ management, the sample was representative of the wards’ nurses (Table 1). We see that there are a lower mean number of patients per nurse in the surgical ward. Management confirmed that this is common during the morning as patients either leave for surgery before the day shift or are admitted for surgery in the afternoon.

**Table 1:** Nurse characteristics

	Surgery	Cardiology	Geriatrics
*N*	10	10	10
Male/female	1/9	2/8	1/9
Mean age, years (range)	40.7 (23–62)	33.5 (24–53)	29.9 (23–41)
Mean work experience, years (range)	13.9 (1–38)	8.75 (0.5–31)	6.0 (1–18)
Mean no. of patients per nurse	2.6	4	3.6

### 4.2 Patients

All 102 patients who were approached by the researchers consented to the observation. For four patients, the nurses deemed the observation as being too emotionally stressful and asked the observer not to follow her into the room, so these patients were not approached by the researcher and were not observed. Five patients were deemed by the nurse to be cognitively impaired, but still suitable for observation. Those patients were asked (if contact was possible) if they objected to the observation, and those who were capable of doing so consented verbally. Two patients were not able to indicate objections, and patients’ families were contacted for permission. From all five families, additional written consent was obtained. On several occasions, the observer was asked to assist, for example to help turn a patient in bed or to help make the bed when the patient was in the bathroom. The observer agreed to do so if it did not interfere with the observation. In two cases, the researcher had to step in during the observation because of patient safety: on one occasion, a patient lost consciousness in a bathroom, and on the other, a patient almost fell.

The 30 transcripts were coded. Twenty‐three were coded by two researchers, while 7 transcripts were single‐coded and checked by a second researcher because of the high level of consensus between the researchers at that point. Open coding resulted in just one extra code: “coordination of care”. All codes of the FoCF were used in the analysis (see Table 2). Coding was done by assigning the relevant fundamental care element to an observation. The assigned code was often a combination of codes which described the situation. For example, for an observation of a nurse washing a patient and talking about the patient experiencing pain, the codes “personal cleansing and dressing” and “comfort” would relate to the patient's physical care, the codes “communication” and “education and information” would relate to the interaction's psychosocial part, and the codes “active listening” and “empathy” would relate to relational skills. Some FoC elements were difficult to discern, as they seem alike and sometimes two elements can describe a situation. This was most obvious in the combinations “active listening” and “being present”, “choice” and “being involved and informed”, and, on occasion, “empathy” and “compassion”.

**Table 2:** Elements of fundamentals of care frequency

Physical	No. codes	Psychosocial	No. codes	Relational	No. codes
Personal cleansing and dressing	212	Communication	151	Active listening	13
Toileting needs	50	Being involved and informed	207	Empathy	43
Eating and drinking	91	Privacy	40	Engaging with patients	44
Rest and sleep	37	Dignity	18	Compassion	46
Mobility	93	Respect	43	Being present and with patients	22
Comfort	106	Education and information	34	Supporting and involving families and carers	5
Safety	226	Emotional well‐being	13	Helping patients to cope	20
Medication management	157	Choice	44	Working with patients to set, achieve and evaluate progressions of goals	3
		Having values and beliefs considered and respected	2		
		Social engagement, company and support	4	Helping patients to stay calm	14
		Feeling able to express opinions and needs without care being compromised	14		
		Having interests and priorities considered and accommodated (where possible)	3		
Coordination of care	203				

## 5 Themes

Analysis of the codes leads to three themes: (a) fundamental care elements, describing what elements were visible during morning care; (b) personalised care versus task‐oriented care, which related to the level of person‐centred fundamental care; and (c) coordination of care, involving the tasks the nurses performed when not at the patient's bedside.

### 5.1 Fundamental care elements

This theme describes what fundamentals of care were observed and what nurses spent most of their time doing. For the majority of the observation periods, the nurses took care of the patients’ physical needs (Table 2). Most of that time was spent on helping or stimulating patients to wash and dress (“personal cleansing and dressing”); preparing, checking and handing out medication (“medication management”); as well as conducting safety checks and measures including vital signs, assessing the patients’ mental state and physical well‐being, and risk reduction activities such as preventing infections or falls (“safety”). Nurses helped patient with mobility, which mainly involved moving from the bed to a chair or the bathroom. The nurses also asked about comfort‐related topics such as pain levels, nausea, body warmth or if the patients were feeling comfortable in their bed or chair. Nurses sometimes asked patients about what or when the patients wanted to eat or drink, but this task was mostly performed by the kitchen staff. Nurses were sometimes observed asking or talking about rest and sleep (quality), and the patients’ toiletry needs or bowel movements. The FoCF’s psychosocial and relational elements were less frequently observed during morning care than its physical elements (Table 2). The most frequently observed psychosocial elements were “communication” and “being involved and informed”, and the most frequent relational elements were “compassion”, “engaging with patients” and “empathy”. The elements that were seldom observed were relational elements: “supporting and involving families and carers” and “working with patients to set, achieve and evaluate progressions of goals”. The psychosocial elements were also infrequently observed: “having values and beliefs considered and respected”, “social engagement, company and support” and “having interests and priorities considered and accommodated (where possible)”.

### 5.2 Personalised care versus task‐oriented care

Observed care ranged from personalised care to more task‐oriented care. All the nurses appeared to be concerned with the patients’ well‐being and would respond in a friendly manner when patients were not feeling well or were distressed. Differences were observed in how this was enacted, with some nurses displaying more empathy, compassion and more active listening. These nurses appeared to be truly present with the patient, and they interacted and connected with the patients’ more explicitly. Where the nurses were observed giving more attention to the patient, an increase in the combinations between the physical, psychosocial and relational elements of fundamental care was observed. An example is illustrated in the observation below, where a nurse was caring for a patient on bed rest in a single bedroom:

The nurse has a calm and friendly way of talking. She explains clearly what she is going to do. The nurse helps the patient with undressing, and says that the patient may help. The patient helps. The patient indicates that something is wrong. The patient cannot find the right word. The patient points to her cheek. The nurse asks “jaw?” The patient says “yes”. The nurse asks if she is in pain, the patient says “yes”. The nurse says that pain in the jaw muscle is common after the operation the patient has had, and explains why. … The nurse asks how the patient experienced the operation yesterday. The patient tells to have experienced little anxiety. The nurse continues washing, the patient is naked, and the nurse covers the patient with towels. The patient tells about the rest of the admission, and the nurse continues washing, frequently seeking eye contact … The nurse helps the patient dress. The patient wants to do parts herself. The nurse encourages the patient by softly saying “well done” during the dressing.(Nurse 5 ward 1)

This nurse incorporated different relational and psychosocial elements in the physical action “personal cleansing and dressing”. She informed the patient of her actions and involved her in the care, letting the patient decide what she could do herself. She was supportive and encouraging when the patient wanted to dress herself. The nurse was attentive to the patient's speech impairment (compassionate and respectful); she picked up on the patient's signals of not being comfortable and acknowledged those signals by telling the patient why she was uncomfortable and not to worry (education and information, helping patient to cope, empathy). She paid attention for possible emotions regarding the operation by bringing the operation up (empathy), and her nonverbal behaviour signalled that she was present with the patient and engaged in active listening. While doing so, she was helping the patient wash, took care of the patient's privacy and comfort by covering her up with towels and helped her get dressed.

However, most nurses were more task‐focused in their interactions. The next observation describes a nurse helping a patient on bed rest to wash in a single bedroom:

The nurse raises the bed to work level and removes the sheets from the patient. The patient is wearing an operation gown without underwear. The nurse removes the intravenous access point on the patient’s foot. The pager goes off, the nurse looks at it, says “I will be right back”, and walks out the door. She leaves the patient with the bed still high and without covers … The nurse comes back a few minutes later and tells the patient she is sorry for having to leave. The patient says that it is no problem and that he was comfortable. The nurse tells that she is there to check the vitals, and to help washing. The nurse checks the vitals. The nurse gives some explanations to me (the observer), not to the patient. The nurse cleans up … The interaction between the nurse and the patient is friendly. Much eye contact and the nurses has a calm appearance. The nurse hands over washcloths to the patient, suggests that the patient washes his face and arms himself, and that she will help with the rest. The patient agrees. During the washing there is little conversation aside from giving instructions, both don’t initiate conversation. The patient makes less eye contact during washing. The patient needs to roll aside to change the bed, the nurse gives instructions. I (the observer) assist with turning upon being asked by the nurse, although the patient appears to be able to turn himself. This was not discussed. The nurse is washing the patient’s buttocks when her pager goes off, room no. 17 is calling. The nurse picks up her phone and calls a colleague to go to the other patient, instructing her on what the question probably will be. The patient is still on his side, the nurse ends the call, and continues washing. There is little communication during the washing.(Nurse 1 ward 1)

Although the nurse had a friendly way of communicating with the patient by making eye contact and having a friendly tone of voice, she was mostly focused on her task which was washing the patient and cleaning the sheets. There were several opportunities to change the interaction: the nurse could have covered the patient up when she needed to leave the room, and not placed a telephone call while washing the patient's privates (respect, dignity, comfort, privacy). Additionally, she gave the observer information when checking the vitals, but this was not directed at informing the patient. There also appeared to be a lack of communication and involvement during the washing. She directed the way that the patient was washed and turned without asking for the patient's wishes or abilities, and did not use this time to connect with the patient. No explicit attention to psychosocial or relational elements could be observed, even though there was plenty of time to do so. The first nurse went through similar tasks and appeared to connect.

Most nurses were observed to attend to their patients’ privacy, especially in rooms with multiple beds. Curtains were closed when a patient needed to get undressed, and for subcutaneous injections and putting on stockings, even when the patients indicated that it was unnecessary. The observer was also asked to stay outside the curtains while some patients were being washed. However, this high regard to privacy did not apply to other nurses who were entering from behind the curtains or into the bathroom. Nurses were often observed entering a privacy‐sensitive situation such as the patient washing, dressing or being on the toilet with little notice. They often walked straight into the room. Patients, however, did not seem bothered by this behaviour. These encounters with fellow nurses mostly concerned discussing general patient care planning. The nurses would also report back on performed tasks or discuss other patients either anonymously (e.g., “your patient in bed 26”) or with mentioning that other patient's name, in the presence of the patient being attended to. One example is this interaction where the observed nurse A was washing an undressed patient in room 4:

Nurse A is washing the patient. Nurse B knocks at the door and walks in. Another patient in room eight is discussed by the nurses regarding comfort and extra medication. Nurse A asks Nurse B if she had the impression that the patient was not comfortable during washing. Nurse B answers that the patient was gasping. She leaves the room. Nurse A resumes washing and starts chatting with the patient, talking about the cold weather and the patient’s dog. A kitchen assistant knocks and walks in. She asks if the family of the other patient in room eight should be offered breakfast. Nurse A tells her to do so because they are holding a vigil for the patient (the patient is dying). “Would you like to wear slippers or shoes?” nurse A asks her patient.(Nurse 4 ward 3)

Most observations started with nurses asking patients questions about safety and essential care‐related topics like pain, nutritional and bowel status, sleep quality and the patients’ vital signs. The patient's responses were documented, and nurses moved on to the next question. Most nurses appeared to have a standard way of checking these items, repeating the same line of questions in the same way with all the patients. However, many nurses did not follow up on the patients’ answer by inquiring further or taking actions to address the topic raised. The questions did not appear to be a deliberate inquiry for further action to be taken, nor were they seen to be incorporated into care planning. Issues were often followed up by referring to a doctor who would come by later, or by offering medication. This lack of follow‐up is visible in the following interaction between a nurse and a cardiac patient:

The nurse asks if the patient has slept well. The patient says that she finally had a good night’s sleep. The nurse says that that is nice. The patient indicates that she is a bit dizzy from the sleep medication. The nurse responds that that is possible. The patient says that a good night’s sleep is worth a lot. The nurse agrees. …

The nurse gave a short response, mainly indicating that she had heard the patient. Dizziness, however, is a common side effect of cardiac medication and might lead to an increased fall risk and general discomfort. The dizziness could have been explored further.

The encounter continues:

(..) The nurse asks if the patient has pain. The patient says to have pain in her mouth. The nurse asks if this pain is new. The patient explains that she had this for a longer time, and that it is caused by a dry mouth. The nurse asks if the patient has water. The patient says no, and that the air is very dry in the ward. The nurse agrees and gets the patient some water. The patient says that the fluid restriction she is on is not helpful for the dry mouth. The nurse says that this is indeed difficult. She asks if the patient can turn off her own apnea machine.(Nurse 6 ward 2)

At first, the nurse's response seems short but appropriate. However, it becomes apparent that the hospital air is dry and that patient has a fluid restriction, causing a dry mouth and thirst. Many patients struggle with complying with fluid restrictions, and offering water is not the appropriate response considering the fluid restriction. The nurse could have explored other options to tackle the dry mouth with the patient and talk about the experienced discomfort to promote therapy adherence.

The specific FoC elements aimed at patient participation in care: “being involved and informed”, “having interests and priorities considered and accommodated (where possible)”, “supporting and involving families and carers” and “working with patients to set, achieve and evaluate progressions of goals”, were observed in a variety of ways. The element of “involving and informing patients in care” was most often observed during personal cleansing and dressing. Nurses asked about the patients’ ability to wash and dress themselves, and discussed with the patients where they needed help. They informed the patients about their actions and gave instructions on what they could do. Nurses focused their communication on explaining their actions to the patients. Patients were rarely informed about what to expect during the rest of the morning or the day, or discharge from the hospital. Most conversations were observed to be one‐sided, with nurses giving information and explaining their actions. Nurses were seen to inquire about the patients’ preferences mostly by giving the patients a choice between two options, such as if they preferred to wash or eat first, if they preferred to take a shower or wash with (prepackaged, heated) washcloths, or if they wished to eat at the bedside or at the table. Patients were seldom observed indicating that they wanted something other than the two options provided. The following elements were rarely observed: “asking about and discussing patients’ needs and goals for recovery”, “empowering patient to ask questions”, “discussing family involvement in care”, “inquiring about a patient's life outside the hospital” and “discussing the patients” need for information on disease or treatment”.

### 5.3 Coordination of care

Observations of the nurses’ actions when they were not at their patients’ bedsides were placed under the theme of coordination of care. These actions mainly concerned communication between nurses about patient care, general ward management such as planning medical rounds or admissions, and asking each other questions. These actions either occurred during unplanned interactions such as meeting each other in the hallway and checking whether they were on schedule, or when nurses deliberately looked for colleagues to either check on what tasks needed to be done or to report back on accomplished tasks. These interactions occurred in hallways and in patient rooms. Another frequently observed interaction was nurses looking and asking around for colleagues to assist them in patient care. Frequently, the nurses who were asked for assistance were in patient care themselves and were interrupted by a colleague stepping into the room. In this example, nurse A has just finished washing and is helping a patient to get dressed:

Nurse B walks into the bathroom and asks for help in the medication room. “I’ll be right there”, responds Nurse A. She continues to help the patient getting dressed. Nurse A puts on the patient's shoes and helps the patient stand up with a walker. Nurse C comes in and says: “can I ask you something?” Nurse A responds: “I’ll be right there.” Nurse C leaves. The patient is helped with sitting down in his wheelchair and is comfortable. Nurse A leaves the room’.(Nurse 1 ward 3)

Nurses were rarely observed being paged away by patients; they were mainly called away by other nurses or other professionals. Usually, they were called to discuss logistical issues such as when to start medical ward rounds, to answer questions (e.g., “do you know how to…”) or to assist with patient care (e.g., washing, transportation, medication checks). Communication with other professionals during care delivery in the morning was observed frequently, with nurses receiving questions, messages or tasks to plan or perform for their patients. Nurses were often handling different tasks or conversations at the same time. One example of this situation is described in the following observation:

Nurse A walks in the hallway. Nurse B inquires if the nurse can go into medical rounds. Nurse A agrees. She walks past the secretary who asks her about a patient being admitted in the afternoon. Nurse A tells her that she is busy, and that she has to do the medical round, and that she also has a multidisciplinary deliberation in the afternoon. Meanwhile, Nurse C asks if she can do anything to help her. Nurse A responds that she still needs to wash the patient in room 1. Nurse C responds that she will help this patient. Nurse A tells Nurse C that she appreciates this and tells the secretary that she can admit the patient at 11:30.(Nurse 5 ward 3)

## 6 DISCUSSION

The results of this study gave in‐depth insights into how fundamental care delivery is enacted. Analysis of the observations led to the identification of three major themes: fundamental care elements, personalised care versus task‐oriented care and coordination of care. The results demonstrated that nurses were focused on physical care delivery in a task‐driven manner and that psychosocial aspects such as addressing patient goals, care planning and patient participation were less frequently observed. Additionally, nurses were often seen interrupting each other's care process, which hindered a person‐centred approach of integrated fundamental care delivery.

### 6.1 Fundamental care elements

All the FoC elements were observed during this study, although some occurred more frequently than others. We observed that nurses spent most of the time taking care of the patients’ physical needs, like washing and dressing and medication management, as well as in performing safety checks such as taking vital signs and filling in safety and comfort checklists. This is unsurprising, as the morning is traditionally a time which revolves around physical care. However, a number of relational and psychosocial FoC elements were rarely observed, and in general they occurred less frequently than the physical elements, confirming a dominant biomedical focus in acute health care (Feo & Kitson, 2016). International literature also indicates that nurses rarely discuss a disease's emotional aspects or explore the patient's feelings actively (Kruijver, Kerkstra, Bensing, & Wiel, 2001), and they rarely report the undertaking of actions to address or improve the patients’ psychosocial needs (Juve‐Udina et al., 2014). Other studies have also reported the difficulty of nursing students with identifying the patients’ psychosocial and relational needs (Jangland et al., 2018). The communication that was observed was often directly related to the physical action or to small talk. Although small talk is important in establishing a nurse–patient relationship, it might also create an atmosphere unsuitable for dealing with emotional or more difficult issues (McCabe, 2004).

### 6.2 Personalised care versus task‐oriented care

Observations within this theme demonstrated that nurses have different ways of building and maintaining professional relationships with patients. Some nurses were observed to have a more person‐centred approach as they used various elements of psychosocial care and relational skills when taking care of a patient's physical needs, confirming that integrated fundamental care delivery is feasible (Feo & Kitson, 2016). Most nurses, however, showed little integrated care, seeming to be focused on task completion and physical care rather than using the time to connect with the patient. Even though this was visible in most physical care aspects, the lack of follow‐up on health status inquiries and picking up on patient cues is the most alarming as it threatens not only the person‐centredness of care, but also the quality and safety of nursing care. Gathering patient information starts the clinical reasoning process, which is an essential feature of healthcare practice. According to Higgs, Burn, and Jones (2001), clinical reasoning in nursing is the process of making professional judgements, by evaluating the quality and contribution of available evidence to enhance problem solving, and by considering the extent to which the evidence available is sufficient to make decisions on diagnosis and treatments options that are relevant to the patient's nursing care requirements (Higgs et al., 2001). Results from the current study, however, indicate that nurses often assess their patient clinical status in a way that seems aimed at task completion. This confirms previous research that nurses perceive themselves as acting in a person‐centred way, but are observed to be centred on routines rather than individual patient assessment and management (Bolster & Manias, 2010). Nurses often inform patients about what they are doing at that moment, but seldom stimulate actual participation or patient involvement in care. The assessments nurses made often appeared not to be followed up by any other actions, thereby hindering the incorporation of patient signs and symptoms into clinical reasoning. If there was follow‐up on the patients’ indicated health status, this was often through referral to medical care, such as medication or a doctor's visit, or to allied health services (e.g., arranging a physiotherapist). This confirms the notion that fundamental care in hospitals is becoming more fragmented (Feo & Kitson, 2016). Next to not actively asking follow‐up questions, observations showed that nurses also often did not pick up or follow up on indirect patient cues that something is worrying them (Suchman, Markakis, Beckman, & Frankel, 1997). This confirms previous findings that about half of all patient cues are responded to with distancing behaviour from nurses (Chan, 2014; Uitterhoeve et al., 2008) even though it is known that following up on patient cues leads to (more) disclosure of concerns by patients (Uitterhoeve et al., 2008). Asking follow‐up questions and picking up on patient cues mean having attention to both physical and psychosocial care. The lack hereof prevents the nurse from progressing from data collection into the process of clinical reasoning. The ability to perform integrated fundamental care and clinical reasoning skills are therefore entwined.

To improve clinical reasoning, nursing curricula have increasingly been focused on teaching clinical reasoning based on nursing diagnosis (Herdman & Kamitsuru, 2017). The use of nursing diagnosis was, however, seldom observed, and also no care plans were made or discussed with patients. This was apparent in the almost complete absence of the codes “working with patients to set, achieve and evaluate progressions of goals”, “having values and beliefs considered and respected” and “having interests and priorities considered and accommodated (where possible)”. Most nurses focused their tasks and communications mainly on physical care and did not explicitly incorporate elements of psychosocial care, even though results show that there was plenty of opportunity within the nurse–patient interaction to do so, confirming that such care does not take up more time or resources (McCabe, 2004). Even though nurses perceive a lack of time as a barrier for patient involvement (van Belle et al., 2018; Tobiano et al., 2015) and integrated care (Conroy, 2018), McCabe (2004) further demonstrated that nurses do not communicate sufficiently in a patient‐centred way even when they have the opportunity to do so, and that patients perceive nurses in general as being more aimed at task completion than on communicating (McCabe, 2004). Physical care then becomes more of an act, rather than an opportunity to connect with a patient as a means to provide patient‐centred care (Feo & Kitson, 2016), even though the quality of the relationship between the nurse and the patient is significantly linked to improved health outcomes such as symptom relief and improvements in clinical and functional status (Safran, Miller, & Beckman, 2006).

According to Kitson (2014), this focus on the patient as a body to do things to, rather than a person to engage with, is reinforced by electronic nursing records that are built on physical care and identify discrete diagnostic and nursing interventions, without demonstrating how these interventions come together to create an integrated care plan and positive experience for the patient (Kitson et al., 2014). The lack of a focus on person‐centred fundamental nursing care in most nurses can also be explained by healthcare systems which are increasingly focused on task completion, outcome evaluation and benchmarking (Feo & Kitson, 2016), and by the pressure on nursing care from shorter admission times and increases in older patient with complex care requirements (World Health Organization, 2015). Australian research found that nurses complete an average of 72.3 tasks per hour and spend only about 37% of their time with patients, which translates to approximately 3.1 hr per 8.5‐hr shift (Westbrook, Duffield, Li, & Creswick, 2011).

### 6.3 Coordination of care

This study's results demonstrate that nurses often interact with each other to discuss patient care, resulting in frequent disturbances during morning care where patient privacy is occasionally threatened. Consistent with this study's findings, other studies have demonstrated that nurses are often interrupted by other nurses seeking help in patient care (Sassaki & Perroca, 2017). During interruptions in care, nurses often did not take their patients’ privacy into consideration. Nurses were observed entering privacy‐sensitive situations, such as the patient washing, being in the toilet and having conversations. The nurses would then demand immediate attention from the attending nurse and often ignored the situation they had entered. The literature indicates that a lack of environmental privacy, impaired health and old age all impact the loss of patient dignity in hospitals, and that this loss threatens the feelings of being comfortable, in control and valued (Baillie, 2009). Baillie (2009) also found that nursing staff was often unaware of how their interactions affect dignity and privacy, which might be strengthened by our findings that the patients often did not seem bothered when the nurses came in or were disturbed, thereby providing few clues to nurses as to whether they were affected. A recent study, however, demonstrated that for older patients, dignity and respect are core values that need to be met in the interpersonal care relationship (Riviere et al., 2019). In previous studies, nurses indicated that they were hindered in having conversations with patients because they were busy and were called away often, and would like to have more time to talk to patients (van Belle et al., 2018). Studies confirm that nursing care is often interrupted, with research indicating that on average there are 2–5.6 interruptions an hour per nurse (Dante et al., 2016; Westbrook et al., 2011). Nurses report lower levels of satisfaction with their performance and higher levels of emotional exhaustion on days with large amounts of workflow interruptions (Pachler et al., 2018). Interruptions also have an effect on patient safety, with nurses make more mistakes when interrupted (Westbrook, Woods, Rob, Dunsmuir, & Day, 2010). Therefore, even though in the current study patients did not seem bothered by nurses being called away, having to divert attention or entering privacy‐sensitive situations, such behaviour can still cause several psychosocial problems, raise safety concerns and affect work satisfaction.

### 6.4 Reflection on fundamentals of care

Although the FoCF was valuable for analysis, it was at times difficult to differentiate between elements like “active listening” and “being present”; “choice” and “being involved and informed”; or “empathy” and “compassion”, as interactions could comprise both elements.

### 6.5 Further research

Findings have shed light on some issues which might prove valuable to further pursue in advancing person‐centred fundamental care. Our findings suggest a direct link between a nurse's ability to provide integrated care and effective clinical reasoning. This, amongst the question what nurse characteristics influence care delivery and the impact of care disturbances, could be further investigated with an experimental study design in which effectiveness of integrated fundamental care in clinical reasoning is assessed.

### 6.6 Limitations

The main limitation of this study is that a certain degree of observer bias might be unpreventable. As all three observers were nurses, it is possible that while observing fellow nurses, their own professional views were reflected in the observations, transcripts and analysis. The researchers took precautions to minimise bias by using an observation guide, by discussing the transcripts with the observed nurses, by reflecting and talking about the experiences with each other and by double coding most of the transcripts. Rigour was enforced by the main researcher co‐observing and co‐transcribing for consistency and by the other observers helping in coding and analysing. Another limitation is the timing, as the observations for the morning interactions do not automatically translate to the rest of the day. The researchers, however, felt that the nurses’ characters and working styles were apparent from the 2.5 hr of observation in the morning, and the focus on how the nurses integrated psychosocial care with relational skills during physical care could be observed well. A strength of the study was the immersion in the nursing care. The aim was to act as much as possible as a passive observant and only intervene in case patient safety was threatened. However, it felt more natural and immersive for the researcher to occasionally assist nurses in their work such as by helping change a bed, when doing so did not disrupt the observation. Many nurses stated that they were quickly used to the presence of the observer, feeling like the observer was a colleague who they were showing around (something they were used to), and did not feel like they were being judged by the researcher writing everything down. The researchers felt that without their own experience as nurses, the observations could not have provided accurate insights into practice, resulting in rich and realistic descriptions of the care provision.

## 7 CONCLUSION

This study demonstrates that few nurses integrated psychosocial care and relational skills in their patients’ physical care. Nurses were often seen to be more task‐oriented in communication, mainly gathering information and telling patients about current tasks. This implies that the care provided was often not patient‐centred and that patient participation was seldom stimulated, even though we saw that there is ample opportunity within an interaction to do so. It is therefore possible to have person‐centred fundamental nursing care in fast‐paced hospital wards, but it needs extensive attention to be improved, with a focus on the integration of psychosocial and relational care into physical care and the clinical reasoning process. Quality of care and person‐centredness can be further improved by attentiveness to patient cues. Frequent disturbances should also be limited as it hinders a person‐centred fundamental care approach.

## 8 RELEVANCE TO CLINICAL PRACTICE

This study gave in‐depth insights into the level of person‐centredness of fundamental nursing care delivery. Nurses were often observed to be rather task‐driven with less attention to integrating the psychosocial and relational aspects of care while attending their patients’ physical needs. However, there were some good examples which indicated that there was sufficient opportunity to do so, making it something that can be improved. Integrating physical, psychosocial and relational care elements in daily practice and in the process of clinical reasoning is needed for high‐quality, person‐centred, fundamental care delivery, in which patients are actively involved in their care.

## CONFLICT OF INTEREST

The authors declare no conflict of interest.
